# Generation of boron-deficiency-tolerant tomato by overexpressing an *Arabidopsis thaliana* borate transporter *AtBOR1*

**DOI:** 10.3389/fpls.2014.00125

**Published:** 2014-04-01

**Authors:** Shimpei Uraguchi, Yuichi Kato, Hideki Hanaoka, Kyoko Miwa, Toru Fujiwara

**Affiliations:** ^1^Biotechnology Research Center, The University of TokyoTokyo, Japan; ^2^Graduate School of Agricultural and Life Sciences, The University of TokyoTokyo, Japan; ^3^Creative Research Institution, Hokkaido University, SapporoJapan

**Keywords:** *AtBOR1*, boron-deficiency, nutrient, tomato, transgenic, transporter

## Abstract

Nutrient deficiency in soil poses a widespread agricultural problem. Boron (B) is an essential micronutrient in plants, and its deficiency causes defects in both vegetative and reproductive growth in various crops in the field. In *Arabidopsis thaliana*, increased expression of a major borate transporter gene *AtBOR1* or boric acid channel gene *AtNIP5;1* improves plant growth under B-deficient conditions. In this study, we examined whether high expression of a borate transporter gene increases B accumulation in shoots and improves the growth of tomato plant, a model of fruit-bearing crops, under B-deficient conditions. We established three independent transgenic tomato plants lines expressing *AtBOR1* using *Agrobacterium*-mediated transformation of tomato (*Solanum lycopersicum* L. cv. Micro-Tom). Reverse transcription-polymerase chain reaction (RT-PCR) analysis confirmed that two lines (Line 1 and Line 2) more strongly expressed *AtBOR1* than Line 3. Wild-type plants and the transgenic plants were grown hydroponically under B-sufficient and B-deficient conditions. Wild-type and Line 3 (weakly expressing transgenic line) showed a defect in shoot growth under B-deficient conditions, especially in the development of new leaves. However, seedlings of Line 1 and Line 2, the transgenic lines showing strong *AtBOR1* expression, did not show the B-deficiency phenotype in newly developing leaves. In agreement with this phenotype, shoot biomass under low-B conditions was higher in the strongly expressing *AtBOR1* line. B concentrations in leaves or fruits were also higher in Line 2 and Line 1. The present study demonstrates that strong expression of *AtBOR1* improved growth in tomato under B-deficient conditions.

## INTRODUCTION

Boron (B) is an essential micronutrient for plants (Warington, 1923). B mediates cross-linking of rhamnogalacturonan-II (RG-II), a component of the pectic polysaccharide, and proper formation of the RG-II dimer is essential for maintenance of the cell wall structure and plasticity (Hu and Brown, 1994; Matoh et al., 1996; O’Neill et al., 1996, 2001). Hence, B is important for root elongation (Kouchi and Kumazawa, 1975), leaf expansion (Kirk and Loneragan, 1988; Huang et al., 1996; Dell and Huang, 1997), viable pollen grain production, and pollen tube elongation (Garg et al., 1979; Cheng and Rerkasem, 1993). B-deficient growth conditions impair vegetative and/or reproductive growth (Dell and Huang, 1997; Shorrocks, 1997), and B-deficiency has been observed in various agricultural soils, which limits crop production globally (Shorrocks, 1997). Application of B fertilizer is one approach to grow crops under B-deficient conditions in the field (Schon and Blevins, 1990), but excess B is also toxic to plants (Nable et al., 1997). A narrow B concentration range exists between deficient and toxic levels for plants, which complicates B fertilizer application (Francois, 1984; Gupta et al., 1985; Schon and Blevins, 1990). Thus, understanding the plant B transport mechanisms is important to improve B nutrition of crops.

Passive diffusion was believed to be the major process of transmembrane B transport prior to the identification of B-transporting molecules (Takano et al., 2008). Physiological evidence of preferential B transport is suggestive of the contribution of transporter molecules to B transport in plants (Dannel et al., 2000; Matoh and Ochiai, 2005; Uraguchi and Fujiwara, 2011). In* Arabidopsis thaliana* roots, AtNIP5;1, a boric acid channel, plays a role in B uptake (Takano et al., 2006). AtBOR1, an efflux B transporter, mediates xylem loading of B (Takano et al., 2002). Another *A. thaliana* B transporter, AtBOR2, contributes to RG-II dimer formation in roots subjected to limited B environments (Miwa et al., 2013). AtNIP6;1, another boric acid channel in *A. thaliana*, mediates preferential B distribution to developing leaves under B-deficiency (Tanaka et al., 2008). These findings suggest that long-distance B delivery and local B distribution/supply are important for plant growth under B-deficient conditions.

Using the identified transporters, transgenic plants tolerant to low-B conditions have been generated by artificially upregulating expression of B transporters in *A. thaliana* plants. Overexpression of AtNIP5;1, a boric acid channel for root B uptake, and/or AtBOR1, an efflux B transporter for xylem loading, improves the vegetative and reproductive growth of *A. thaliana* under B-deficient conditions (Miwa et al., 2006; Kato et al., 2009). The generation of B-deficiency-tolerant *A. thaliana* plants suggests that upregulating B-transporter expression can improve the growth of crops under B-deficiency.

*AtBOR1* homologous genes have been isolated from crops such as rice (Nakagawa et al., 2007; Tanaka et al., 2013), grapevine (Pérez-Castro et al., 2012), wheat (Leaungthitikanchana et al., 2013), and *Brassica napus* (Sun et al., 2012). In addition, B-deficiency-tolerant cultivars of rice, maize, and wheat show increased *BOR1* transcript levels (Leaungthitikanchana et al., 2014). This indicates that *BOR1* homologs are highly conserved in crops and can be used to improve B-deficiency-tolerance. However, the effects of overexpressing *BOR1* on B-deficiency-tolerance have not been examined in crops.

In this study, we introduced the *AtBOR1* gene into a tomato model cultivar (*Solanum lycopersicum* L. cv. Micro-tom) and established *AtBOR1*-overexpressing tomato plants. Tomato is a model plant of fruit-bearing crops. The occurrence of B-deficiency in tomato cultivation fields has been reported in several countries from Europe, Asia, South America, and Africa (Shorrocks, 1997). We examined the growth and B accumulation in these plants subjected to different B conditions. Two independent lines strongly expressing *AtBOR1* showed normal leaf development, even under B-deficient conditions, and higher B accumulatioin in shoots/fruits compared with non-transgenic tomato plants. These results suggest that upregulating B-transporter expression may improve the growth of fruit-bearing crops under B-deficient conditions.

## MATERIALS AND METHODS

### PLANT MATERIAL AND CONSTRUCTION

Seeds of tomato cv. Micro-Tom, a dwarf tomato cultivar (Scott and Harbaugh, 1989) were obtained from Kazusa DNA Research Institute (Chiba, Japan) and University of Tsukuba (Ibaraki, Japan). A plasmid carrying the CaMV 35S RNA promoter (P35S):*AtBOR1* was constructed for the transformation of tomato plants based on hygromycin selection. The P35S:*AtBOR1* fragment of pTF469 (Miwa et al., 2006) was amplified by polymerase chain reaction (PCR) using the primers 5′-CACCAGATTAGCCTTTTCAATTTCAG-3′ and 5′-GATCTAGTAACATAGATGACACCGC-3′. The amplified fragment was subcloned into pENTR/D-TOPO (Invitrogen, Carlsbad, CA, USA). P35S:*AtBOR1* was then subcloned into pMDC99 (Curtis and Grossniklaus, 2003) using the LR recombination reaction. The resulting plasmid was named pHH104.

### PLANT TRANSFORMATION

pTF469 and pHH104 were used for transformation of tomato plants to obtain plants expressing *AtBOR1*. Kanamycin (Wako Pure Chemicals, Osaka, Japan) and hygromycin (Roche Diagnostics, Basel, Switzerland) were used for selecting the transformants carrying pTF467 and pHH104, respectively. *Agrobacterium*-mediated transformation of Micro-Tom was performed as described previously (Sun et al., 2006) with minor modifications. Briefly, for hygromycin selection, 5 mg/L of hygromycin was added to the callus induction and shoot induction media. Antibiotic selection was not applied during the rooting step.

### PCR-BASED CONFIRMATION OF THE T-DNA INSERTION

Genomic DNA was extracted from the leaf (about 5 mg) as described previously (Kasajima et al., 2004). Obtained DNA was used as template to confirm T-DNA integration into the genome by PCR. Primers specific to *AtBOR1* and NOS-terminator in the T-DNA were designed as 5′-CGTGGAAACCGTTCCATTC-3′ and 5′-GCCAAATGTTTGAACGATCGG-3′, and were used to amplify the T-DNA fragment. Tomato EST SGN-E341940 was selected as a gene with homology to *AtACTIN2* (At3G18780) and was referred to as the *Actin-like* gene. The primers specific to *Actin-like* were 5′-TGTTGCTATTCAGGCTGTGC-3′ and 5′-AATCACGACCAGCAAGATCC-3′.

### REVERSE TRANSCRIPTION (RT)-PCR ANALYSIS

Seeds of non-transgenic and transgenic plants (T_1_) were germinated on vermiculite and grown for 14 days (22°C, 16 h light/8 h dark). T-DNA insertion was then examined by PCR as described above. PCR-positive transgenic plants and non-transgenic plants were transferred to MGRL hydroponic solution (Fujiwara et al., 1992) containing 100 μM boric acid. The solution was replaced weekly. Fourteen days after transfer, roots of the plants were harvested for RNA extraction. Total RNA was extracted using the RNeasy Plant Mini Kit (Qiagen, Hilden, Germany). The RNase-Free DNase Kit (Qiagen) was used to eliminate DNA contamination. Reverse transcription was conducted using PrimeScript RT reagent Kit (Takara Bio, Shiga, Japan). PCR was performed using obtained cDNA as template. Primers 5′-AATCTCGCAGCGGAAACG-3′ and 5′-TGGAGTCGAACTTGAACTTGTC-3′ were used for *AtBOR1* expression analysis. The expression of the *Actin-like* gene was also examined as a control using the primers described above.

### PLANTS CULTURED UNDER DIFFERENT B SUPPLIES

Plants were incubated at 25°C under a 16 h light/8 h dark cycle. Seeds of non-transgenic and transgenic plants (T_1_) were sown on vermiculite and grown for 19 days, after which the T-DNA insertion was examined by PCR as described above. PCR-positive transgenic plants and non-transgenic plants were transferred to MGRL hydroponic solution supplemented with 0.1 or 100 μM boric acid. The solution was renewed twice a week for the 0.1 μM boric acid treatment and once a week for the 100 μM boric acid treatment.

### DETERMINATION OF BORON CONCENTRATIONS IN PLANT TISSUES

To determine the shoot B concentration, whole shoots were harvested after 20 days of hydroponic culture and then dried at 60°C for at least 3 days. After determination of total shoot dry weight, samples were acid-digested and subjected to inductively coupled plasma mass spectrometry (ICP-MS; SPQ-9000, Seiko Instruments Inc., Chiba, Japan) for quantifying the B concentration (Takano et al., 2002). Plants were also grown hydroponically until fruit ripening and harvest. After drying and acid digestion, B concentrations in the fruits were determined by ICP-MS.

### STATISTICAL ANALYSES

To verify the statistical significance of differences among the lines, the data were analyzed using the Student’s *t*-test (*p* < 0.05).

## RESULTS

### GENERATION OF TRANSGENIC TOMATO EXPRESSING *AtBOR1*

**FIGURE 1:**
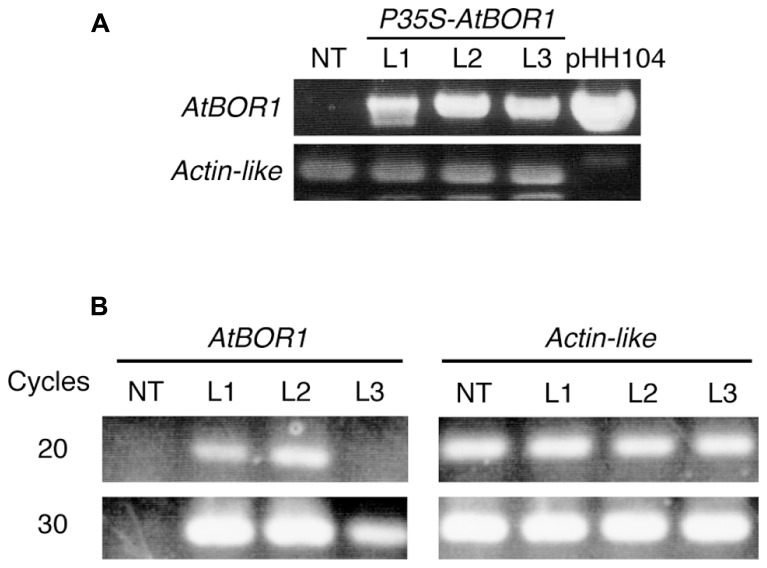
**Establishment of transgenic tomato expressing *AtBOR1***. **(A)** Genomic DNA was extracted from non-transgenic (NT) and regenerated T_0_ plants (L1, L2, and L3). PCR was performed using genomic DNA as a template with primers specific to the T-DNA or tomato *Actin*-like gene. pHH104 was also used as a control for T-DNA. **(B)** Total RNA was extracted from the roots of non-transgenic plants (NT) and transgenic T1 plants (L1, L2, and L3). Semiquantitative RT-PCR was performed using primers specific to *AtBOR1* or the *Actin*-like gene.

After several batches of transformation, 11 independent candidate lines were obtained. Among them, three lines showed good fertility and more than 100 seeds were obtained from each line. These three lines were used for the following experiments. Genomic DNA was extracted from a leaf of regenerated plants (T_0_). The T-DNA integration into the genome was examined on these plants by PCR analysis using the specific primers for T-DNA (**Figure 1A**). All tested regenerated T_0_ plants showed a band at the expected size. No band corresponding to the T-DNA fragment was obtained from non-transgenic plants using PCR. The *Actin-like* gene was used as a positive control and all plants (including non-transgenic plants) showed a band at the expected size. These results indicated that the regenerated plants contained the T-DNA insertion in their genome. These three lines were named L1, L2, and L3.

Reverse transcription-polymerase chain reaction analysis was conducted to investigate whether the introduced *AtBOR1* gene was expressed in these transgenic lines (**Figure 1B**). *AtBOR1* expression was clearly observed after 20 cycles of PCR in L1 and L2, whereas no signal was detected in non-transgenic plants and L3. A relatively weak signal of *AtBOR1* was obtained from L3 after 30 cycles of PCR compared to L1 and L2, but not from non-transgenic plants. These results suggest that introduced *AtBOR1* is expressed relatively strongly in L1 and L2 compared to L3.

### TRANSGENIC TOMATO PLANTS WITH STRONG EXPRESSION OF *AtBOR1* DID NOT SHOW B-DEFICIENCY SYMPTOMS UNDER LOW-B GROWTH CONDITIONS

**FIGURE 2:**
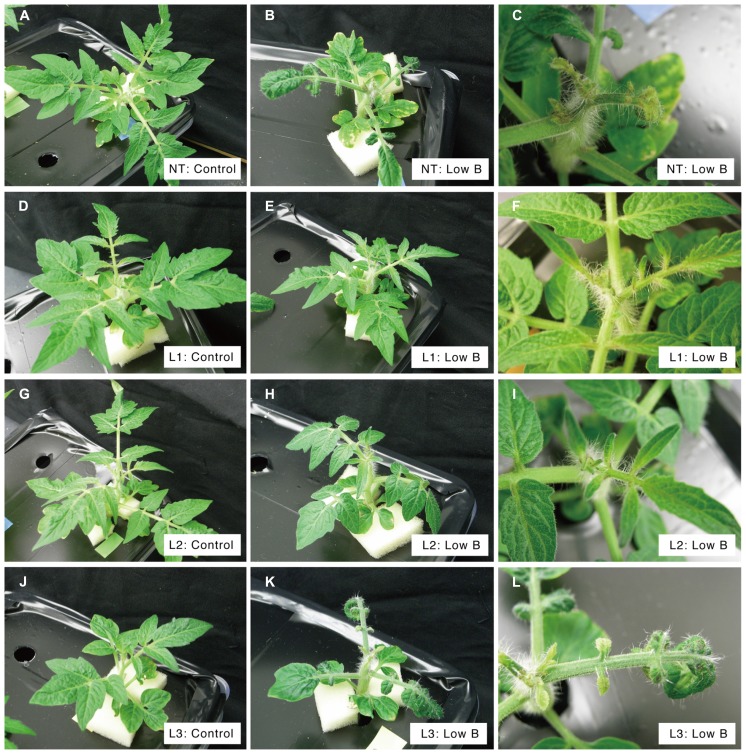
**Shoot development of transgenic tomato plants expressing *AtBOR1* under B-deficiency and sufficiency**. Non-transgenic (NT) plants **(A–C)**, transgenic plants L1 **(D–F)**, L2 **(G–I)**, and L3 **(J–L)** were grown for 15 days with a hydroponic solution supplied containing 100 μM (control) or 0.1 μM (low-B) boric acid. Representative plants under control **(A,D,G,J)** and low-B **(B,E,H,K)** conditions and their developing leaves under low-B conditions **(C,F,I)** are shown.

To investigate the effect of heterologous *AtBOR1* expression on growth of tomato plants under B-deficiency, non-transgenic plants and three transgenic T_1_ lines were grown hydroponically in the presence of 0.1 or 100 μM boric acid. Experiments were performed with at least four replications and representative individuals are shown in **Figure 2**. Under B-sufficient conditions (100 μM B), shoot growth and development were normal in all tested lines (**Figures 2A,D,G,J)**, although L3 showed relatively small shoots (**Figure 2J**) compared to other samples (**Figures 2A,D,G**). Phenotypic differences among lines were clear under the low-B conditions (0.1 μM B). In non-transgenic plants under the low-B conditions, shoot growth was retarded compared to the control (**Figures 2A,B**). Curly leaves were observed and development of newly growing leaves was often inhibited by the B-deficient treatment (**Figure 2C**). These phenotypes were also observed in L3 subjected to the low-B treatment (**Figures 2K,L**), but not observed in L1 and L2 plants (**Figures 2E,F,H,I**). In L1 and L2, development of new leaves was normal, even under the low-B condition (**Figures 2F,I**). These results indicated that heterologous expression of *AtBOR1* increases tolerance of tomato plants to B-deficiency stress.

### ENHANCED BORON ACCUMULATION IN TOMATO PLANTS EXPRESSING *AtBOR1* UNDER THE LOW-B GROWTH CONDITION

**FIGURE 3:**
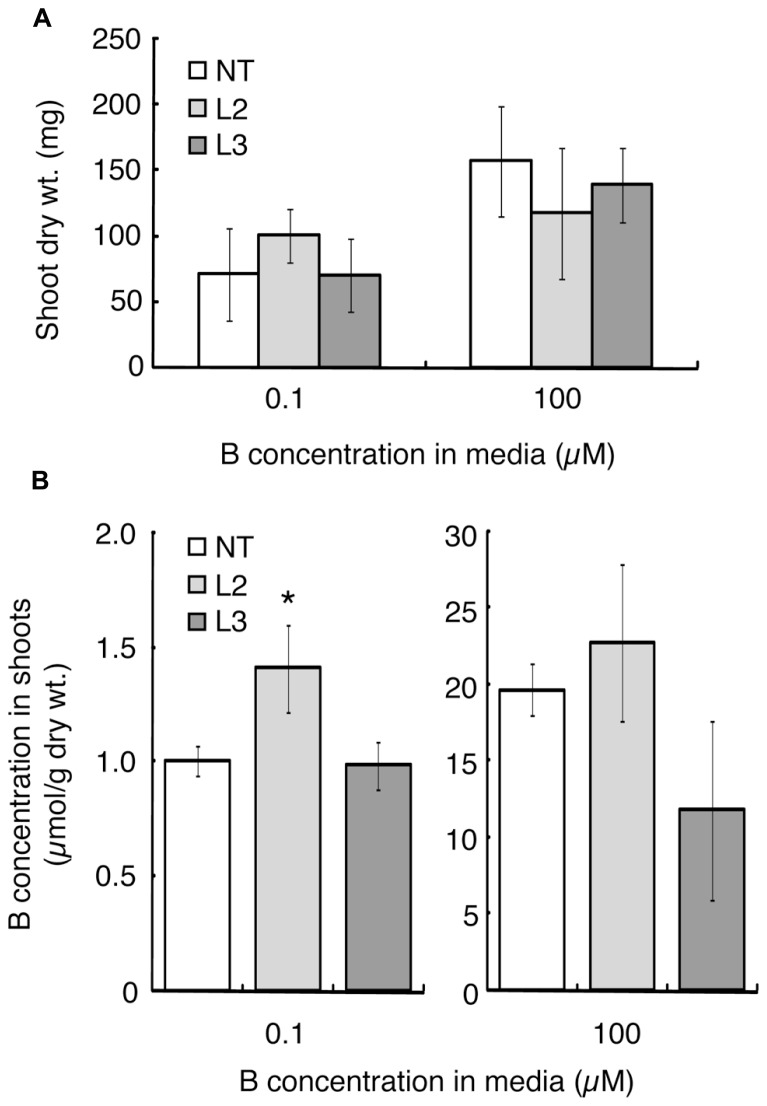
**Shoot dry weights and B concentrations in transgenic tomato plants expressing *AtBOR1* under B-deficiency and sufficiency**. Non-transgenic (NT) and transgenic (L2 and L3) plants were grown for 20 days in a hydroponic solution containing 0.1 or 100 μM boric acid. Shoot dry weight **(A)** and B concentration in shoots **(B)** were measured. Values represent the means ± standard deviation (*n* = 3–4). An asterisk indicates significant differences from non-transgenic plants (Student’s *t*-test, *p* < 0.05).

Shoot dry weight and B concentration were measured after 20 days of hydroponic culture under the B-sufficient and B-deficient conditions. L1 was not tested in this experiment due to the limited seed numbers. Shoot dry weight of L2, which showed improved B-deficiency-tolerance (**Figures 2H,I**), was slightly higher than those of the non-transgenic plants and L3, although not significantly (**Figure 3A**). This tendency was not observed in the 100 μM boric acid treatment.

The B concentration in shoots was compared among the lines (**Figure 3B**). Under the low-B treatment, shoot B concentration was 1.4-fold higher in L2 than in non-transgenic plants and L3. When plants were supplied with 100 μM boric acid, B accumulation in shoots did not differ significantly between the non-transgenic plants and L2. L3 tended to accumulate less B than non-transgenic plants.

**FIGURE 4:**
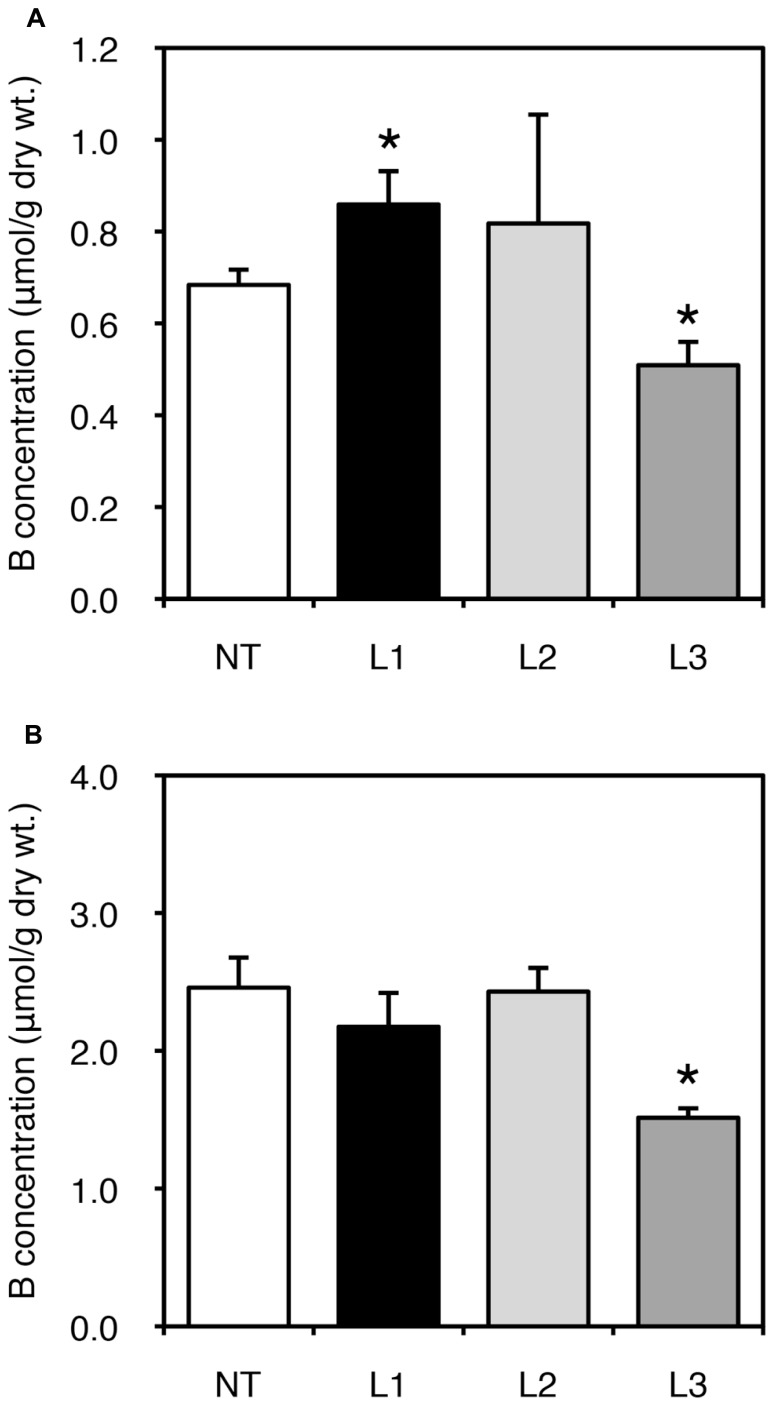
**Fruit B concentrations in transgenic tomato plants expressing *AtBOR1* under B-deficiency and sufficiency**. Non-transgenic (NT) and transgenic (L1, L2, and L3) plants were grown in a hydroponic solution supplied with 0.1 or 100 μM boric acid until fruit setting. Fruits were harvested from plants grown under 0.1 μM **(A)** or 100 μM **(B)** B treatment. B concentrations in the fruits were measured using ICP-MS. Values represent the means ± standard deviation (*n* = 3–4). An asterisk indicates significant differences from the non-transgenic plants (Student’s *t*-test, *p* < 0.05).

We also examined fruit yields of non-transgenic and transgenic plants. In all lines, low-B treatment impaired fruit yield; however, no consistent result was obtained due to the large variation among plants (data not shown). We next measured B concentration in fruits harvested from the plants grown under B-sufficient and B-deficient conditions (**Figure 4**). For the low-B treatment, the fruits of L1 accumulated significantly higher B than non-transgenic plants (**Figure 4A**). Fruit B concentration under the B-deficient condition appeared higher in L2 than in non-transgenic plants, although the difference was not significant. This tendency of an increased B concentration in L1 and L2 was not observed under B-sufficient conditions (**Figure 4B**). The B concentrations in fruits of L3 was relatively lower than in non-transgenic plants under both B treatments. These results suggest that B accumulation in shoots and fruits is enhanced in the stronger *AtBOR1* expression lines.

## DISCUSSION

As discussed in a recent review (Schroeder et al., 2013), regulation and manipulation of plant membrane transporters can be used to improve crop production under various soil-derived stresses such as aluminum toxicity and nutrient deficiency. B-deficiency occurs in various fields globally (Shorrocks, 1997). Based on the molecular mechanisms of B transport, we generated *A. thaliana* plants tolerant to low-B conditions by upregulating B-transporter genes (Miwa et al., 2006; Kato et al., 2009). Increased expression of *AtNIP5;1* and/or *AtBOR1* significantly improved vegetative and reproductive growth of *A. thaliana* with limited B supply. Since the concentration range between B-deficiency and toxicity is rather narrow in many plants, improper B fertilization can lead to an excess dosage and negatively affect crop growth (Francois, 1984; Gupta et al., 1985; Schon and Blevins, 1990). Therefore, as established in *A. thaliana*, molecular breeding of crops with enhanced B-transport activity is a promising approach to address B-deficiency (Miwa et al., 2006; Kato et al., 2009). However, no attempt has been made to establish B-deficiency-tolerant crops by enhancing *BOR1* expression.

In this study, tomato was selected as a test plant because it is a common model of fruit-bearing crops. The occurrence of B-deficiency has been reported for tomato cultivation in many countries (Shorrocks, 1997). Genotypic variation of B-uptake activity and B-deficiency-tolerance among tomato cultivars has been observed (Brown and Jones, 1971; Bellaloui and Brown, 1998), which indicates that enhancement of B-transport efficiency confers B-deficiency-tolerance in tomato. B-deficiency symptoms of tomato plants are represented by shoot growth inhibition, curly and yellowish leaves of young seedlings, and defects in quality fruit setting during the reproductive growth stages (Johnston and Fisher, 1930; Brown and Jones, 1971; Yamauchi et al., 1986).**

In the present study, wild-type tomato plants showed retarded whole shoot growth, curled leaves, and abnormal and poor development of new leaves in the presence of 0.1 μM boric acid (**Figures 2A–C and 3A**). These phenotypes are typical B-deficiency symptoms reported in early tomato seedlings, suggesting that our experimental condition was suitable for evaluating the effects of *AtBOR1* overexpression in young tomato plants grown under limited B availability. We performed several batches of transformation experiments and obtained three independent transgenic tomato lines carrying P35S-*AtBOR1* (**Figure 1A**) with substantial seed yields. Expression analysis of introduced *AtBOR1* in these lines demonstrated that L1 and L2 were strongly expressing lines and L3 was a rather weakly expressing line (**Figure 1B**). These strongly expressing lines did not show severe B-deficiency symptoms, which were observed in non-transgenic plants as well as in weakly expressing L3 (**Figure 2**). Furthermore, shoot and fruit B accumulation under the low-B conditions significantly increased in L1 and L2, respectively, compared to non-transgenic plants, but not in L3 (**Figures 3B and 4A**). These results suggest that enhanced expression of a B transporter improves the root-to-shoot translocation of B in tomato under low-B availability, which contributes to maintaining proper shoot development. Since the effect of *AtBOR1* expression on yield remains unclear due to the considerable variation observed under our hydroponic conditions, this should be further examined with an improved cultivation system and moderate B-deficiency conditions.

Optimal B concentrations in culture media are narrow, and overdose of B leads to increased B accumulation in plant tissues and plant growth inhibition (Francois, 1984; Gupta et al., 1985; Schon and Blevins, 1990). Excess B increases DNA damage, which may cause B toxicity in plants (Sakamoto et al., 2011). Concentrations of B in shoot and fruits under B-sufficient conditions were similar between the non-transgenic and *AtBOR1*-expressing plants (**Figures 3B and 4B**). Growth under the 100 μM boric acid condition was comparable among the non-transgenic and transgenic plants (**Figures 2 and 3A**), suggesting that overexpression of *AtBOR1* has a negligible adverse effect on tomato young seedlings under B-sufficient conditions. In *A. thaliana* roots, AtBOR1 protein levels are regulated by post-translational mechanisms, and protein degradation is promoted under B-sufficient conditions through ubiquitination of AtBOR1 (Takano et al., 2005; Kasai et al., 2011). This regulation of AtBOR1 likely prevents B overaccumulation in plants. In fact, AtBOR1-overexpressing *A. thaliana* accumulates wild-type levels of B under excess B treatments (Miwa et al., 2006). In this study, we examined the effects of heterologous *AtBOR1* expression in tomato, but similar mechanisms may regulate AtBOR1 levels in tomato under B-sufficient conditions.

## CONCLUSION

In the present study, we established transgenic tomato plants tolerant to B-deficient environments by upregulating a B-transporter gene. Since B-deficiency is a serious issue in various crop productions, our findings suggest that application of AtBOR1 or its ortholog to crop breeding may improve growth under B-deficient cultivation conditions.

## Conflict of Interest Statement

The authors declare that the research was conducted in the absence of any commercial or financial relationships that could be construed as a potential conflict of interest.
